# Long-term trends in the incidence of hospital-acquired carbapenem-resistant *Enterobacterales* and antimicrobial utilization in a network of community hospitals in the Southeastern United States from 2013 to 2023

**DOI:** 10.1017/ice.2024.173

**Published:** 2025-01

**Authors:** Tark Kim, Rebekah W. Moehring, Nicholas A. Turner, Elizabeth Dodds Ashley, Linda Crane, Polly Padgette, Valerie C. Payne, Linda Roach, Brittain Wood, Deverick J. Anderson

**Affiliations:** 1 Division of Infectious Diseases, Department of Internal Medicine, Soonchunhyang University Bucheon Hospital, Bucheon, Republic of Korea; 2 Duke Center for Antimicrobial Stewardship and Infection Prevention, Durham, NC, USA; 3 Duke Antimicrobial Stewardship Outreach Network, Durham, NC, USA; 4 Duke Infection Control Outreach Network, Durham, NC, USA

## Abstract

**Background:**

Carbapenem-resistant *Enterobacterales* (CRE) are an urgent threat to healthcare, but the epidemiology of these antimicrobial-resistant organisms may be evolving in some settings since the COVID-19 pandemic. An updated analysis of hospital-acquired CRE (HA-CRE) incidence in community hospitals is needed.

**Methods:**

We retrospectively analyzed data on HA-CRE cases and antimicrobial utilization (AU) from two community hospital networks, the Duke Infection Control Outreach Network (DICON) and the Duke Antimicrobial Stewardship Outreach Network (DASON) from January 2013 to June 2023. The zero-inflated negative binomial regression model was used owing to excess zeros.

**Results:**

126 HA-CRE cases from 36 hospitals were included in the longitudinal analysis. The pooled incidence of HA CRE was 0.69 per 100,000 patient days (95% confidence interval [95% CI], 0.57–0.82 HA-CRE rate significantly decreased over time before COVID-19 (rate ratio [RR], 0.94 [95% CI, 0.89–0.99]; *p* = 0.02), but there was a significant slope change indicating a trend increase in HA-CRE after COVID-19 (RR, 1.32 [95% CI, 1.06–1.66]; *p* = 0.01). In 21 hospitals participating in both DICON and DASON from January 2018 to June 2023, there was a correlation between HA-CRE rates and AU for CRE treatment (Spearman’s coefficient = 0.176; *p* < 0.01). Anti-CRE AU did not change over time, and there was no level or slope change after COVID.

**Conclusions:**

The incidence of HA-CRE decreased before COVID-19 in a network of community hospitals in the southeastern United States, but this trend was disrupted by the COVID-19 pandemic.

## Introduction

Carbapenem-resistant *Enterobacterales* (CRE) infection is associated with high mortality and cost; 26%–44% of deaths in patients with CRE infection are attributable to carbapenem resistance.^1^ In 2015, CRE infections in the United States cost hospitals $275 million and resulted in the loss of 8,841 quality-adjusted life years.^2^ Because of these adverse outcomes, CRE has become a major public health concern, posing significant challenges to infection prevention and control in healthcare settings. This threat is further amplified in low-resource settings such as community hospitals, where resources and specialized infection control expertise might be limited.

The corona virus disease 2019 (COVID-19) pandemic created new challenges for infection prevention and control, leading to increased healthcare-associated infections (HAI) and antimicrobial-resistant organisms. For example, in a large cross-sectional analysis involving 182 hospitals in the United States between 2020 and 2022, the incidences of major HAI and methicillin-resistant *Staphylococcus aureus* (MRSA) bacteremia were higher in the COVID-19 population.^3^ These results were also consistent when national- and state-level standardized infection ratios were calculated for each HAI and quarter by dividing the number of reported infections by the number of predicted infections, calculated using 2015 national baseline data.^4^ Similarly, the Center for Disease Control and Prevention (CDC) published a special report in 2022, documenting a 35% increase in CRE infections in US hospitals in 2020.^5^ The impact of COVID-19 on CRE and the use of antibiotics for CRE treatment in community hospitals, however, has not been well described.

The correlation of prior antimicrobial utility (AU) with the development of antimicrobial resistance has been well known, and prior AU data can be used for evaluating the risk of antimicrobial resistance.^6^ Unlike other antibiotics, the treatment options for CRE are extremely limited,^7^ so anti-CRE AU data may serve as an indirect indicator for CRE incidence in a setting of limited CRE surveillance. Additionally, the proportions of each anti-CRE regiment can be indirectly used to evaluate the impact of antimicrobial stewardship programs by whether CRE is treated as recommended in guidelines. For example, in Korean national AU data, a notable upward trend of colistin use in primary care hospitals suggested a need for antimicrobial stewardship programs in these hospitals.^8^

In a prior summary of data from 16 community hospitals, we documented a 5-fold increase in CRE from Jan 2008 to Dec 2012.^9^ The current study summarizes an updated analysis of 10 years of surveillance data on the incidence of hospital-acquired CRE (HA-CRE) from 2013 to 2023 and anti-CRE AU data from 2018 to 2023 within a network of community hospitals, including time periods before and after the onset of the COVID-19 pandemic from 2013 to 2023.

## Methods

### Study design

This retrospective study includes three analyses that utilize prospectively collected surveillance data from two community hospital networks, the Duke Infection Control Outreach Network (DICON) and the Duke Antimicrobial Stewardship Outreach Network (DASON). Briefly, DICON has been assisting community hospitals in the southeastern US for over 25 years, providing infection prevention data analysis and metrics, access to experts in infection control, opportunities to share successful programs, and extensive educational initiatives related to infection prevention.^10^ As part of network activities, surveillance data on HA-CRE are systematically and prospectively compiled. DASON has been assisting community hospitals in the southeastern US for over 10 years, providing data collection, analysis, feedback, educational resources, and expert consultation for antimicrobial stewardship activities.^11^

**Figure 1.**
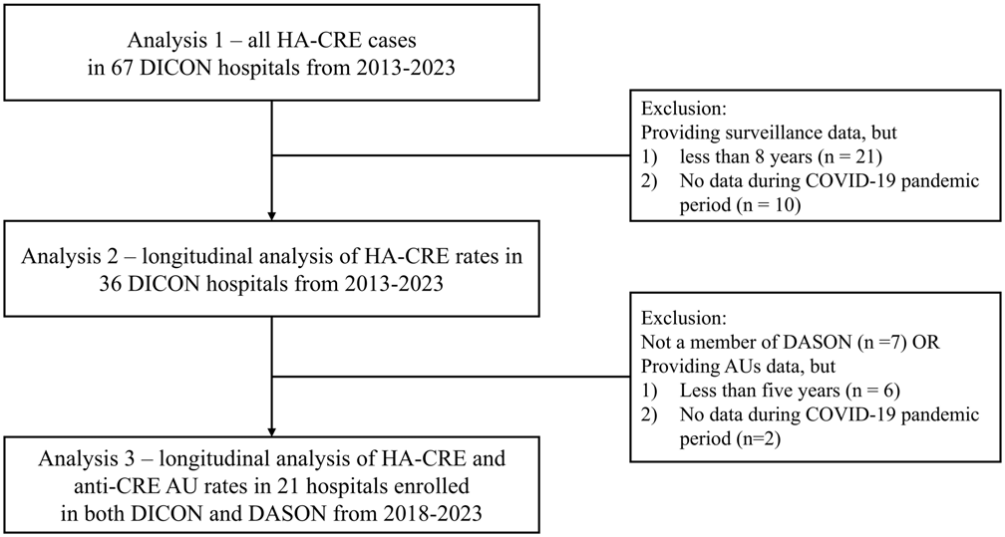
Study design–selection of hospitals for three analyses. AU, antimicrobial utilization; COVID-19, corona virus disease 2019; DASON, Duke Antimicrobial Stewardship Outreach Network; DICON, Duke Infection Control Outreach Network; HA-CRE, hospital-acquired carbapenem-resistant *Enterobacterales*.

The study design and hospital selection criteria for the three analyses are summarized in Figure 1. All analyses included surveillance data on HA-CRE collected from DICON community hospitals in North Carolina, South Carolina, Virginia, and Georgia from January 2013 to June 2023. Analysis 1 was a descriptive analysis of all HA-CRE cases identified among 67 DICON hospitals. Analysis 2 was a longitudinal analysis of HA-CRE data from 36 hospitals that submitted data for 8 or more years including the COVID-19 period from January 2020 to June 2023 during the 10-year study period. Analysis 3 included HA-CRE and antimicrobial utilization (AU) data from 21 hospitals participating in both DICON and DASON for five or more years between January 2018 and June 2023.

### Surveillance data, patients, and definition

Local infection preventionists (IPs) performed prospective surveillance for CRE using standardized protocols in all hospitals. CRE was defined using CDC definitions based on phenotypic susceptibility.^12^ The IPs collected and entered data on patients with CRE isolates into a centralized database, including the following variables: infection versus colonization, year of birth, sex, ethnicity, date of hospital admission, previous admissions to the same hospital during the preceding year, specimen collection date and type, types of infections, and whether the admission was from home or another healthcare facility. IPs also routinely entered monthly patient days for each hospital.

All CRE-positive cultures from the surveillance database during the study period were reviewed for inclusion. If an individual patient had multiple hospitalizations during which CRE was detected, only the first isolate and admission were registered in the surveillance. “Hospital-acquired” was defined as identification that occurred on or after calendar day 3 of hospital admission.^13^ Based on this definition, only HA-CRE cases were included in the analysis. To determine infection versus colonization, IPs examined the medical record and spoke to the primary healthcare providers to evaluate for signs or symptoms consistent with infection. The definitions in the DICON protocols used to identify urinary tract infection, bloodstream infection, pneumonia, and ventilator-associated events were the same as those of NHSN.^13^ All IPs were trained on NHSN definitions, which allowed for standardized surveillance protocols to be followed.

Quarterly rates of AU for systemic anti-CRE regimens in each hospital, including ceftazidime-avibactam, meropenem-vaborbactam, imipenem-relabactam, cefiderocol, tigecycline, colistin, polymyxin B, eravacycline, and plazomicin as well as oral fosfomycin for treating the urine were retrieved from the DASON database.

### Statistics

Descriptive statistics were used to characterize the epidemiology of HA-CRE in the 67 study hospitals. Incidence rates were calculated as the number of patients with HA CRE per 100,000 patient days. AU was calculated as the days of antimicrobial therapy (DOT) per 1,000 patient days. The chi-square test or Fisher’s exact test was used for categorical variables, and the Mann–Whitney U-test was used for continuous variables.

Owing to a large proportion of zero values and overdispersion of data, we conducted an interrupted time series (ITS) analysis using segmented zero-inflated negative binomial (ZINB) regression to assess HA-CRE rate and anti-CRE AU trends pre- and post-COVID-19 pandemic, as previously described.^14^ ZINB regression handles excess zeroes in data by splitting the model into two components: the zero model (a logistic regression predicting 0 counts) and a count model (traditional negative binomial regression). There was no autocorrelation in HA-CRE incidence in a Durban–Watson statistic (DW = 1.955, *P* = 0.19), and there was positive autocorrelation in the anti-CRE AU trend (DW = 0.82252, *P* < 0.01). Due to concerns of autocorrelation and clustering bias, we did segmented generalized estimating equation regression analysis, and the results were not different from those of the ZINB model (data not shown). Since the results of ZINB have limitations in providing an intuitive understanding of data, we used a generalized linear regression model for the overall trend analysis. The first case of COVID-19 was detected in February 2020, and COVID-19 was widely spread through the United States in March 2020.^15^ Data on the HA-CRE rate was collected quarterly, so the interruption point was defined as April 1, 2020. The Spearman correlation coefficient was used to measure the correlation between pooled HA-CRE rates and AU. All significance tests were two-tailed, and *P* values < .05 were considered significant. All statistical analyses were performed with R software for Windows, version 4.4.0 (R Foundation for Statistical Computing Vienna, Austria).

## Results

### Microorganisms and epidemiologic characteristics of HA-CRE

In total, 152 HA-CRE cases from 67 community hospitals were recorded in our surveillance database during the study period; 33 hospitals reported no HA-CRE cases. The pooled HA-CRE rate for total hospitals was 0.56 per 100,000 patient days (95% confidence interval [95% CI], 0.47–0.62). Epidemiologic characteristics are described in Table 1. *Klebsiella pneumoniae* (n = 85, 55.9%) was the most common HA-CRE microorganism, followed by *Enterobacter cloacae* (n = 25, 16.2%) and *Escherichia coli* (n = 24, 15.8%). Of 152 cases, 96 (63.2%) met the criteria for infection, while 56 (36.8%) were considered as colonization. Urinary tract infection (n = 46, 33.3%) was the most common type of infection, followed by bloodstream infection (n=17, 11.2%), ventilator-associated event (n = 9, 5.9%), non-ventilator pneumonia (n = 7, 4.5%) and others (n = 17, 11.2%). All variables were not statistically different between cases with infection and colonization.

**Table 1.** Epidemiological characteristics of hospital-acquired carbapenem-resistant *Enterobacterales* in a network of 67 community hospitals in southeastern United States from January 2013 to June 2023

Characteristics	Number (%) of patients		
	Total (n = 152)	Infection (n = 96)	Colonization (n = 56)
**Organism**			
*Klebsiella spp.*	101 (66.4)	64 (66.7)	37 (66.1)
*K. pneumoniae*	85 (55.9)	51 (53.1)	34 (60.7)
*K. aerogenes* (formerly *Enterobacter aerogenes*)	12 (7.9)	10 (10.4)	2 (3.6)
*K. oxytoca*	4 (2.6)	3 (3.1)	1 (1.8)
*Enterobacter cloacae*	25 (16.2)	13 (13.5)	12 (21.4)
*Escherichia coli*	24 (15.8)	18 (18.8)	6 (10.7)
*Proteus mirabilis*	1 (0.7)	1 (1.0)	0
*Morganella morganii*	1 (0.7)	0	1 (1.8)
**Demographics**			
Median age (IQR, year-old)	68 (57 –75)	68 (57 –75)	68 (60 –81)
Male	85 (55.9)	58 (60.4)	27 (48.2)
**Race**			
African American	48 (32.2)	26 (27.1)	23 (41.1)
Caucasian	53 (34.9)	32 (33.3)	21 (37.5)
American Indian	1 (0.7)	1 (1.0)	0
Asian	1 (0.7)	0	1 (1.8)
Hispanic	1 (0.7)	1 (1.0)	0
Other	4 (2.6)	2 (2.1)	2 (3.6)
Unknown	43 (28.3)	34 (35.4)	9 (16.1)
**Admitted from**			
Home	73 (48.0)	49 (51.0)	24 (42.9)
Home health	3 (2.0)	0	3 (5.4)
Hospital	15 (9.9)	7 (7.3)	8 (13.3)
Nursing home	24 (15.8)	12 (12.5)	12 (21.4)
Other extended care facility	5 (3.3)	1 (1.0)	4 (7.1)
Other	1 (0.7)	0	1 (1.8)
Unknown	29 (19.1)	25 (26.0)	4 (7.1)

### HA-CRE incidence longitudinal evaluation

Thirty-six hospitals with 126 HA-CRE cases were recorded during 18,362,161 cumulative patient days during the study period and were included in the longitudinal analysis. The median bed size of this group was 214 beds (interquartile range, 129–331 beds). The follow-up period was 11 years in 24 hospitals, 10 years in 2 hospitals, 9 years in 8 hospitals, and 8 years in 2 hospitals. No HA-CRE cases were reported in 14 hospitals. Smaller hospitals (less than 200 beds) were more likely to report no cases of HA-CRE compared to larger hospitals (more than 200 beds; 66.7% vs. 19.0%; *P* = 0.01). The median number of HA-CRE cases at each hospital was one case during the surveillance period (range, 0–19 cases). The pooled incidence of HA-CRE was 0.69 per 100,000 patient days (95% CI, 0.57–0.82).

**Figure 2.**
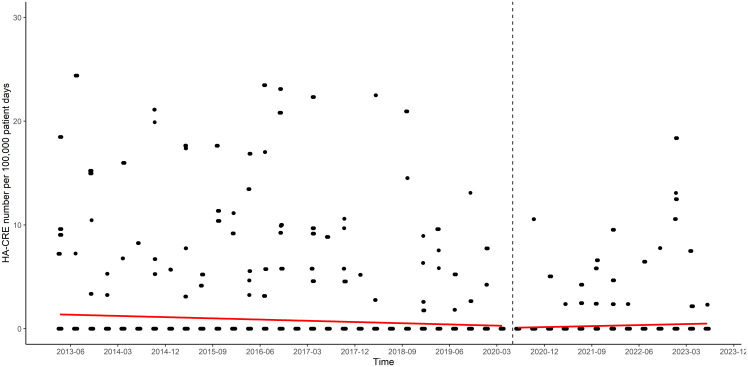
Scatter plots of quarterly HA-CRE rates of 36 southeastern community hospitals in the United States. Two outlier points were omitted from visual space of the graph: 99.50 per 100,000 patient days at the fourth quarter in 2015 and 47.88 per 100,000 patient days at the first quarter in 2020. Dashed line means the onset of corona virus disease 2019 pandemic as the interruption. Red line means linear regression line before and after interruption. HA-CRE, hospital-acquired carbapenem-resistant *Enterobacterales*.

HA-CRE rates decreased over time during the study period (a quarterly decrease of 0.031 per 100,000 patient days; rate ratio [RR], 0.97 [95% CI, 0.95–0.99]; *P* < 0.001; Figure 2). However, important changes were observed when comparing rates before and after the onset of the COVID-19 pandemic. Pooled, unadjusted quarterly HA-CRE rates were 0.67 per 100,000 patient days (95% CI, 0.54–0.81) before COVID-19 and 0.43 per 100,000 patient days (95% CI, 0.29–0.62) after COVID-19, respectively. In the count model of ZINB regression, HA-CRE rate significantly decreased over time before COVID-19 (RR, 0.94 [95% CI, 0.89–0.99]; *P* = 0.02), but there was a significant slope change indicating an increase after COVID-19 (RR, 1.32 [95% CI, 1.06–1.66]; *P* = 0.01; Table 2). In the zero model of ZINB regression, the HA-CRE rate did not change over time before COVID-19 (*P* = 0.63), and there was no significant slope (*P* = 0.50) and level change (*P* = 0.18) after COVID-19 (Table 2).

**Table 2.** Segmented zero-inflated negative binomial regression for the trend of hospital-acquired carbapenem-resistant *Enterobacterales* rate before and after corona virus disease 2019 in 36 southeastern community hospitals in the United States

Regression model	Variables^*^	Coefficient	Standard error	Incidence rate ratio (95% CI)	*P*-value
NB (the count model)	Time before COVID-19	–0.062	0.027	0.94 (0.89–0.99)	0.02
	Level change of time variable after COVID-19	–1.355	1.057	0.26 (0.03–2.05)	0.20
	Slope change of time variable after COVID-19	0.279	0.114	1.32 (1.06–1.66)	0.01
BL (the zero model)	Time before COVID-19	–0.046	0.095	0.96 (0.76–1.15)	0.63
	Level change of time variable after COVID-19	–2.46	3.677	0.09 (0.00006–115.35)	0.50
	Slope change of time variable after COVID-19	0.44	0.328	1.55 (0.82–2.95)	0.18

95% CI, 95% confidence interval; BL, binary logistic; COVID-19, corona virus disease 2019; NB, negative binomial.

* Time was quarterly evaluated.

### A trend of AU for CRE treatment

Twenty-one hospitals were included in Analysis 3 (Figure 1). In this analysis, 88 cases of HA-CRE were included. The overall AU for all anti-bacterial agents was 808.80 DOT per 1,000 patient days from 2018 to 2023. The overall AU for CRE treatment was 1.53 DOT per 1,000 patient days during the period.

Ceftazidime-avibactam was the most commonly used anti-CRE agent (0.66 DOT per 1,000 patient days). Other agents were used less frequently: fosfomycin, 0.41 DOT per 1,000 patient days; eravacycline, 0.22 DOT per 1,000 patient days; tigecycline 0.21 DOT per 1,000 patient days; cefiderocol, 0.12 DOT per 1,000 patient days; colistin IV 0.07 DOT per 1,000 patient days; meropenem-vaborbactam, 0.07 DOT per 1,000 patient days; imipenem-relabactam 0.03 DOT per 1,000 patient days; polymyxin B IV 0.02 DOT per 1,000 patient days. Plazomicin was not utilized in these study hospitals during the study period.

**Figure 3.**
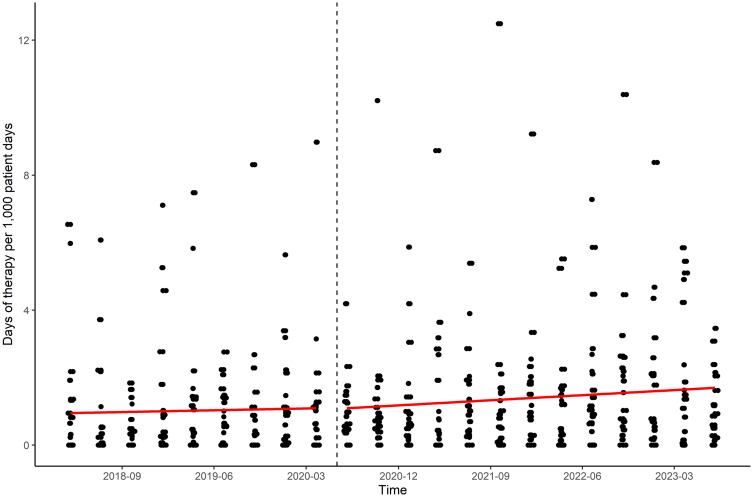
Scatter plots of quarterly AU for CRE treatment of 21 southeastern community hospitals in the United States. Dashed line means the onset of corona virus disease 2019 pandemic as the interruption. Red line means linear regression line before and after interruption. AU, antimicrobial utilization; CRE, carbapenem-resistant *Enterobacterales*.

Anti-CRE AU varied by hospital and quarter (Figure 3). Among the quarterly anti-CRE AU observations, 25.8% (118/456) was zero. Anti-CRE agents were not utilized in one (4.8%) and these were utilized only in two quarters in two (9.6%) hospitals. Overall, anti-CRE AU increased over time during the study period (a quarterly increase of 0.037 DOT per 1,000 patient days; RR, 1.04 [95% CI, 1.01–1.06]; *P* = 0.006). The cumulative AU was 1.27 days of therapy per 1,000 patient days (95% CI, 1.22–1.33) before COVID-19 and 1.66 days of therapy per 1,000 patient days (95% CI, 1.62–1.70) after COVID-19 (*P* < 0.001). In both the count model (RR, 0.99 [95% CI, 0.91–1.07]; *P* = 0.72) and the zero model (RR, 0.85 [95% CI, 0.68–1.18]; *P* = 0.19) of ZINB regression analysis, anti-CRE AU did not change over time. The changes of anti-CRE AU in level and slope after COVID-19 were not significant in both models (supplement table 1). There was a correlation between HA-CRE rates and AU for CRE treatment (Spearman’s coefficient = 0.176; *P* < 0.01; supplement figure 1).

## Discussion

Our study presents the decade-long trend in the incidence of HA-CRE in community hospitals across the Southeastern United States from January 2013 to June 2023. We observed a decreasing trend of HA-CRE incidence in community hospitals participating in DICON prior to the onset of COVID-19. However, this downward trend was not sustained after the onset of COVID-19. Among the subgroup of hospitals for which we analyzed both CRE and AU data, we observed a correlation between HA-CRE and anti-CRE AU. These data provide longitudinal epidemiologic data on HA-CRE and AU and highlight the possible role of IPC and antimicrobial stewardship networks in community hospitals.

In the United States, the percentage of carbapenem resistance in *Enterobacterales* and the incidence of CRE were decreasing for several years before the COVID-19 pandemic. National Health Safety Network (NHSN) data including *E. coli*, *K. pneumoniae*, and *Enterobacter* showed decreasing trend of carbapenem resistance rates from 4.3% (95% CI, 3.9%–4.7%) in 2011 to 2.4% (95% CI, 2.2%–2.6%) in 2019].^16^ In a report published by CDC using electrical health records from more than 700 geographically diverse acute care hospitals, the burden of CRE cases was estimated as 11,800 cases in 2012 and 13,100 cases in 2017, respectively.^17^ However, this decreased to 11,900 cases in 2019 compared to 2017.^5^ In a recent report of data from seven hospitals in Colorado, Georgia, Maryland, Minnesota, New York, Oregon, and Tennessee that continuously participated in CRE surveillance during 2016–2020, the crude CRE incidence rate was statistically significant decreased from 7.51 per 100 000 in 2016 to 6.08 in 2020 (RR, 0.76 [95% CI, 0.70–0.83]), although only the 23% decrease in 2019 (RR, 0.77 [95% CI, 0.61–0.98]) was significant for HA-CRE.^18^ Our data additionally highlight the nationwide decrease of CRE in the aspects of HA-CRE in community hospitals.

Our study showed overall low numbers of HA-CRE in our network of community hospitals. It is unclear whether participation in a network supporting IPC activities of community hospitals led to this low number of HA-CRE. Assuming that patients with a lower risk of CRE acquisition are admitted to smaller community hospitals, this observation may be related to patient case mix and selection bias. In NHSN CRE surveillance data, central line-associated bloodstream infection was the most common type of CRE infection, and a decrease in carbapenem resistance was only prominent in catheter-associated urinary tract infections.^16^ Unlike NHSN data, urinary tract infection was the most common type of CRE infection in our study hospitals. Further well-designed studies should be performed to quantitatively estimate the effect of infection prevention interventions and strategies on HAI and antimicrobial resistance in community hospitals.

Our data showed that the decreasing trend of HA-CRE in our network of community hospitals was not sustained during and after the COVID-19 pandemic. This disrupted trend of HA-CRE rates has also been reported in nationwide data. Although overall estimated CRE cases were stable, HA-CRE cases increased from 3,400 cases in 2019 to 4,300 cases in 2020.^5^ In a recent systematic review of 30 studies on global antimicrobial resistance epidemiology, the incidence density of CRE generally increased, although these changes varied according to CRE species.^19^ The exact cause of increasing AMR rates during the COVID-19 pandemic period was likely multifactorial, including such issues as shortages of isolation facilities, shortages in personal protective equipment, changes in the use of contact precautions, overload of healthcare workers, and increased use of antibiotics. In addition, this increase was clearly impacted by superimposed infection/colonization in patients with COVID-19. A cohort study of 148 hospitals in the United States corroborated that major HAIs, including CLABSI and CAUTI, and MDROs increased in proportion to COVID-19 surges during the pandemic.^3^ Microbiology data obtained from 81 participating hospitals showed that rates of MDROs, including MRSA, vancomycin-resistant *Enterococcus*, and Gram-negative organisms, were also significantly associated with COVID-19 surges.^20^ Unlike previous studies, our study provides data on HA-CRE in 2022 and 2023; to date, we still cannot conclude whether the negative impact of the COVID-19 pandemic on HA-CRE will persist or wane.

We also evaluated AU for CRE-focused treatment because these data may represent the clinician’s judgment of a patient’s risk for CRE or due to a microbiologic diagnosis of CRE. Although the HA-CRE rate and AU for CRE showed a statistically significant correlation, the strength of the correlation was weak. Antibiotics used to treat CRE can be used in other clinical scenarios, such as drug-resistant *Pseudomonas* infections, thereby potentially weakening the correlation. Also, it is not possible to determine anti-CRE regimens used for whether HA-CRE or community-onset CRE. Our study showed the limit of AU use as an indirect measure of the HA-CRE incidence. However, AU data for CRE treatment can still be helpful in estimating the adoption and use of newer agents for multidrug-resistant pathogens. For example, Clancy et al. used prescription data to compare intravenous use of colistin and polymyxin B, the longstanding first-line antibiotics against CRE infections, with that of newer agents such as ceftazidime-avibactam, meropenem-vaborbactam, and plazomicin.^21^ Our AU data suggest that newer agents such as ceftazidime/avibactam were successfully adopted for CRE treatment in a network of community hospitals with little to no use of higher toxicity, older agents.

Our study has limitations. First, outliers of HA-CRE rates in quarters when HA-CRE in-hospital outbreaks occurred may distort the overall trend. However, results were unchanged after excluding two outlier points in sensitivity analyses (data not shown). Second, data on COVID-19 infection at the time of HA-CRE acquisition were not collected. These data may help determine whether this disrupted trend of HA-CRE rates after COVID-19 resulted from superimposed infection or collateral effects of changes in healthcare delivery during the pandemic. Third, while standardized protocols and definitions for surveillance were used, our data may suffer from misclassification bias; individual hospital IPs may have utilized different strategies for case identification. As a result, our data likely represent the minimum burden of CRE in our community hospitals. Fifth, data on molecular testing for carbapenemases were not collected. Finally, time series data repeat observations within the hospital, and they technically violate the independence assumption. In this case, either generalized estimating equations (GEE) or mixed effects models are usually used to account for the violation of independence. Despite this, we chose ZINB as the statistical model for dealing with the problem of excess zeros, and these results were not different from those of the segmented GEE model (data not shown).

In conclusion, the incidence of HA-CRE decreased during the recent 10 years in community hospitals participating in the IPC network in the Southeastern United States, though this trend flattened and was not sustained after the onset of the COVID-19 pandemic. Ongoing surveillance will be needed to determine if this changing trend will resolve or serve as a harbinger for worsening trends.

## Supporting information

Kim et al. supplementary material Kim et al. supplementary material

## Supplementary material

The supplementary material for this article can be found at https://doi.org/10.1017/ice.2024.173.
