# Supplementary material for: Long-term trends in the incidence of hospital-acquired carbapenem-resistant Enterobacterales and antimicrobial utilization in a network of community hospitals in the Southeastern United States from 2013 to 2023
Source: Infect Control Hosp Epidemiol. 2024 Dec 3;46(1):43–9. doi: 10.1017/ice.2024.173 (PMC11717479; doi:10.1017/ice.2024.173)
Supplement: Kim et al. supplementary material [file S0899823X24001739sup001.docx]

**Supplement table 1. Segmented zero-inflated negative binomial regression for the trend of antimicrobial utilization against carbapenem-resistant *Enterobacterales* before and after coronavirus disease 2019 in 21 southeastern community hospitals in the United States**

| Regression model | Variables^*^ | Coefficient | Standard error | Rate ratio (95% CI) | *P*-value |
| --- | --- | --- | --- | --- | --- |
| NB (the count model) | Time before COVID-19 | -0.015 | 0.041 | 0.99 (0.91 – 1.67) | 0.72 |
|  | Level change of time variable after COVID-19 | -0.071 | 0.254 | 0.93 (0.57 – 1.54) | 0.78 |
|  | Slope change of time variable after COVID-19 | 0.051 | 0.046 | 1.05 (0.96 – 1.15) | 0.28 |
| BL (the zero model) | Time before COVID-19 | -0.157 | 0.120 | 0.85 (0.68 – 1.08) | 0.19 |
|  | Level change of time variable after COVID-19 | -0.707 | 1.51 | 0.49 (0.03 – 9.52) | 0.64 |
|  | Slope change of time variable after COVID-19 | 0.093 | 0.212 | 1.10 (0.72 – 1.66) | 0.66 |

95% CI, 95% confidence interval; BL, binary logistic; COVID-19, coronavirus disease 2019; NB, negative binomial

^*^ Time was quarterly evaulated

**Supplementary figure 1. A trend of pooled quarterly HA-CRE rates and AU for CRE Tx of 21 southeastern community hospitals in the United States.**

**
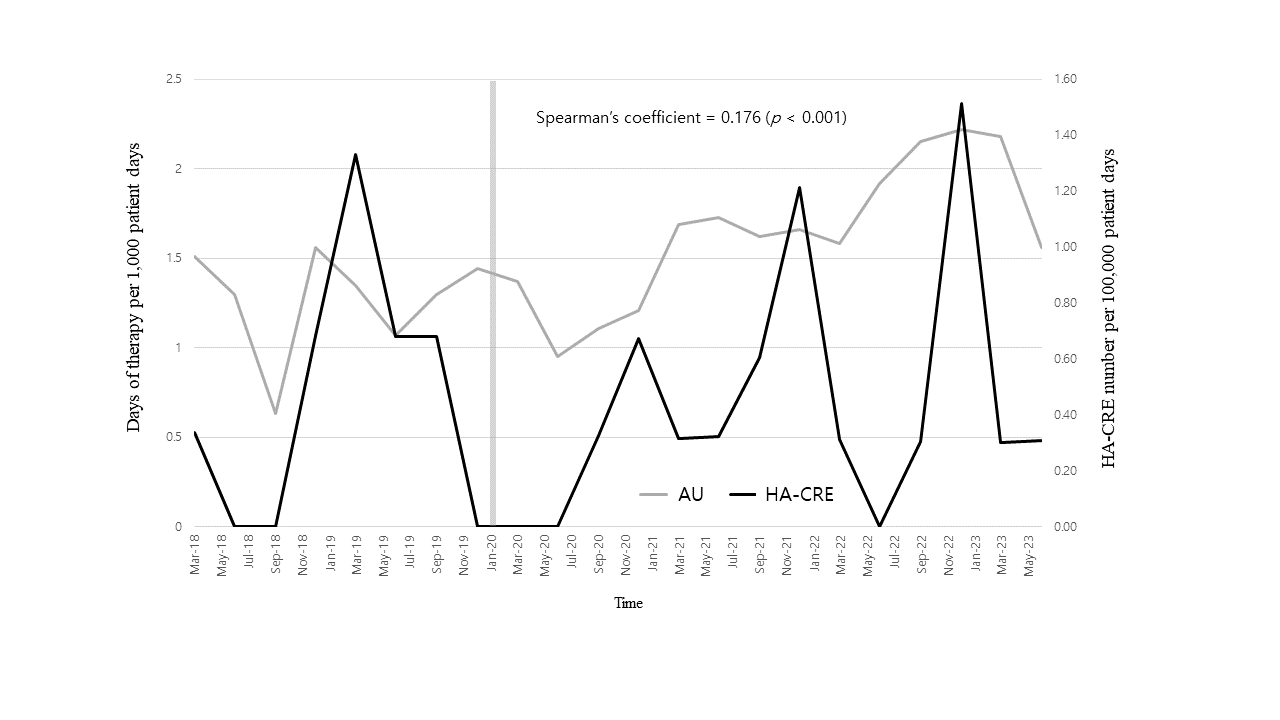
**
